# Dl‐3‐N‐butylphthalide promotes angiogenesis and upregulates sonic hedgehog expression after cerebral ischemia in rats

**DOI:** 10.1111/cns.13104

**Published:** 2019-02-19

**Authors:** Pan‐Ting Zhou, Li‐Ping Wang, Mei‐Jie Qu, Hui Shen, Hao‐Ran Zheng, Li‐Dong Deng, Yuan‐Yuan Ma, Yu‐Yang Wang, Yong‐Ting Wang, Yao‐Hui Tang, Heng‐Li Tian, Zhi‐Jun Zhang, Guo‐Yuan Yang

**Affiliations:** ^1^ Shanghai Jiao Tong Affiliated Sixth People’s Hospital Neuroscience and Neuroengineering Research Center Med‐X Research Institute and School of Biomedical Engineering Shanghai Jiao Tong University Shanghai China; ^2^ Department of Neurology, School of Medicine, Ruijin Hospital Shanghai Jiao Tong University Shanghai China; ^3^ Department of Neurology, The Affiliated Hospital of Qingdao University Qingdao University Qingdao China; ^4^ Department of Neurology, School of Medicine, Zhongshan Hospital Fudan University Shanghai China; ^5^ Department of Rehabilitation Medicine, The Affiliated Hospital of Qingdao University Qingdao University Qingdao China

**Keywords:** angiogenesis, dl‐3‐N‐butylphthalide, growth factor, ischemic stroke, sonic hedgehog

## Abstract

**Introduction:**

Dl‐3‐N‐butylphthalide (NBP), a small molecule drug used clinically in the acute phase of ischemic stroke, has been shown to improve functional recovery and promote angiogenesis and collateral vessel circulation after experimental cerebral ischemia. However, the underlying molecular mechanism is unknown.

**Aims:**

To explore the potential molecular mechanism of angiogenesis induced by NBP after cerebral ischemia.

**Results:**

NBP treatment attenuated body weight loss, reduced brain infarct volume, and improved neurobehavioral outcomes during focal ischemia compared to the control rats (*P* < 0.05). NBP increased the number of CD31^+^ microvessels, the number of CD31^+^/BrdU^+^ proliferating endothelial cells, and the functional vascular density (*P* < 0.05). Further study demonstrated that NBP also promoted the expression of vascular endothelial growth factor and angiopoietin‐1 (*P* < 0.05), which was accompanied by upregulated sonic hedgehog expression in astrocytes in vivo and in vitro.

**Conclusion:**

NBP treatment promoted the expression of vascular endothelial growth factor and angiopoietin‐1, induced angiogenesis, and improved neurobehavioral recovery. These effects were associated with increased sonic hedgehog expression after NBP treatment. Our results broadened the clinical application of NBP to include the later phase of ischemia.

## 1 INTRODUCTION

Stroke is one of the leading causes of morbidity, disability, and mortality in the world.^1^ Currently, thrombolysis and endovascular interventional are the most effective treatment strategies in the early stage after ischemic stroke and both target blood restoration in the ischemic area. However, the time window for this treatment is limited, and the hemorrhage risk is high.^2^ The development of a new strategy to increase blood supply to the ischemic area is necessary and urgent.

Cell death induced by poor blood flow and perfusion is the main pathology of ischemic stroke.^3^ For the treatment of ischemic stroke, blood restoration could be a potential therapeutic strategy.^4^ Angiogenesis was demonstrated to be an important event to increase blood flow after cerebral ischemia. Previous reports showed that angiogenesis occurred 7 and 14 days after ischemia in rodents and occurred in the human brain as early as 3 days following ischemic insult.^5^, ^6^, ^7^ In addition, clinical observation demonstrated a strong correlation between neovascularization and functional recovery, which suggested that newly formed vessels contributed to behavioral outcomes after ischemic stroke.^8^

Dl‐3‐N‐butylphthalide (NBP) is a small molecule drug used in clinical ischemic stroke in China, and Phase II clinical trials in acute ischemic stroke were approved by the FDA in 2016.^9^, ^10^ NBP was first extracted from celery seeds and showed protective effects against ischemic stroke. The beneficial effect of NBP is attributed to its anti‐inflammatory, anti‐apoptotic, antioxidant, pro‐mitochondrial function, and pro‐neurogenesis properties, etc.^9^, ^11^, ^12^, ^13^ Previous studies found that NBP could protect the brain microvascular endothelial cells against oxygen‐glucose deprivation (OGD) through increasing HIF‐1α and Bcl‐2 expression. NBP could also promote angiogenesis through the increased expression of angiogenic growth factors.^14^ However, how NBP promotes angiogenesis in the brain is unknown.

Sonic hedgehog (Shh) is a morphogen that controls skeletal and vascular development in the embryo and is also reactivated during adult repair processes.^15^ Shh holds great therapeutic potential for biotechnological and biomedical approaches aiming to enhance tissue regeneration or to replace damaged tissues.^16^ The role of Shh in the development of functional vasculature in embryonic stages has been observed, and Shh is one of the key elements that guides angiogenesis and vasculogenesis.^17^, ^18^ According to the current literature, Shh controls the expression of several families of growth factors involved in neovascularization and vessel maturation, and it functions upstream of angiogenic growth factors.^19^, ^20^ The expression of Shh and its 45‐kD uncleaved precursor was evaluated in human and mouse astrocytes in vitro and in vivo, and an ~19‐kD active form was identified in the astrocyte‐conditioned medium (CM).^21^

NBP treatment promotes angiogenesis by increasing angiogenic growth factor expression.^22^, ^23^ Additionally, Shh guides angiogenesis and controls the expression of several families of growth factors.^19^, ^20^ Therefore, in the present study, we studied the expression of angiogenic growth factors and Shh in vivo and in vitro after NBP treatment to explore whether the pro‐angiogenesis effect of NBP occurs through the upregulation of Shh in astrocytes.

## 2 MATERIALS AND METHODS

### 2.1 Middle cerebral artery occlusion in rats

Adult male Sprague Dawley rats (n = 90) weighing 250‐300 g were used. This study was carried out in accordance with the recommendations of the Animal Research: Reporting of in vivo Experiments (ARRIVE) guidelines and with government approval by the State Agency for Nature, Environment and Consumer Protection North Rhine‐Westphalia. The protocol was approved by the Institutional Animal Care and Use Committee (IACUC) of Shanghai Jiao Tong University, China.

The surgical procedure for transient middle cerebral artery occlusion (MCAO) was previously described.^24^ Rats were anesthetized by ketamine/xylazine (100/10 mg/kg, Sigma‐Aldrich, St Louis, MO) and positioned supinely on a heating pad (RWD Life Science, Shenzhen, China) to maintain the temperature at 37 ± 0.5°C. With an operating microscope (Leica, Wetzlar, Germany), the left common carotid artery, internal carotid artery, and external carotid artery were carefully isolated. A nylon monofilament suture of 4‐0 (Covidien, Mansfield, MA) coated with silica was gently inserted from the external carotid artery stump to the internal carotid artery and occluded the origin of the middle cerebral artery for 90 minutes. Successful occlusion was characterized as the reduction in cerebral blood flow down to 20% of baseline, which was verified by laser Doppler flowmetry (Moor Instruments, Axminster, Devon, UK).

### 2.2 Experimental design and NBP treatment

NBP (purity > 96%) was obtained from Shijiazhuang Pharmaceutical Group Co. Ltd. (Shijiazhuang, China) and diluted in vegetable oil. All rats were randomly divided into three groups: the NBP‐treated MCAO group, the oil‐treated MCAO group, and the sham group. NBP (80 mg/kg) or equal volume of vegetable oil was administered by gavage once a day from 1 day to 14 days after MCAO.^22^, ^25^, ^26^ 5‐Bromo‐2′‐deoxyuridine (BrdU, Sigma, San Louis, MO) was injected intraperitoneally twice a day at a concentration of 50 mg/kg for 3 days before sample collection.

### 2.3 Behavioral tests

Adult SD rats underwent neurobehavioral tests after MCAO and were assessed by an investigator who was blinded to experimental groups. Rats were trained for 3 days before MCAO and measured at 1, 3, 7, and 14 days after MCAO. The rotarod test was used to evaluate fore and hind limb motor coordination and balance. Rats were allowed to adapt on the rod for 1 minute, after which the velocity of the rod was slowly increased from 15 to 40 revolutions per minute over 5 minutes. The time of rats stayed on the accelerating rotating rod was recorded and analyzed.^27^ Modified neurological severity scores (mNSS) of the animals were graded on a scale of 0 to 14 and included assessment of motor skills, sensory tests, reflex tests, and balance skills (normal score, 0; maximal deficit score, 14).^28^ The elevated body swing test (EBST) was used to assess asymmetric motor behavior, in which rats were suspended vertically by the base of tail 10 cm over the testing surface. The number of turns in either direction (left or right) was recorded when rats swung their heads laterally to the left or right and turned their upper body >10 degrees to either side, and each rat was underwent 20 trials.^29^

### 2.4 Brain infarct volume measurement

The brain infarct volume was evaluated by Cresyl violet staining. Brains were frozen in −80°C isopentane after being transcardially perfused with saline and fixed by 4% paraformaldehyde. Coronal sections (20 μm thick) were cut from the anterior commissure to the hippocampus and mounted onto slides. The distance between each section was 400 μm. The infarct area of each section was delineated by ImageJ (NIH, Bethesda, MD) and determined by subtracting the Cresyl violet stained area in the ipsilateral hemisphere from the whole area of the contralateral hemisphere. The infarct volume was calculated as

$$V=\sum_{1}^{n}\left[\left(S_{n}+\sqrt{S_{n}\times S_{n+1}}+S_{n+1}\right)\times \frac{h}{3}\right],$$

where *h* = 0.4 mm, which represents the distance between each section, and *S* represents the infarct area (mm^2^) in each brain section.^30^

### 2.5 Immunostaining

Sections were blocked for 1 hour in 10% bovine serum albumin at room temperature after treatment with 0.3% Triton‐100 in PBS for 15 minutes to permeabilize the cell membrane. Then, the cells were incubated with primary antibodies against CD31 (1:300 dilution, R&D systems, Minneapolis, MN), GFAP (1:1000 dilution, Millipore, Temecula, CA), MAP2 (1:200 dilution, Millipore), Iba‐1 (1:1000 dilution, WAKO, Osaka, Japan), and Shh (1:200 dilution, Santa Cruz Biotechnology, Santa Cruz, CA) at 4°C overnight. Each section was washed and incubated with appropriate secondary antibodies (Invitrogen, Carlsbad, CA). The primary antibodies were replaced by PBS for the negative controls. BrdU staining was performed as previously described.^31^ For each section, four brain regions were randomly selected from the area of interest and visualized under a fluorescence microscope with a 20× or 40× objective lens (Leica, Solms, Germany). The selected regions of each section are designated by the black boxes, and the gray area represents the infarcted brain region after ischemic stroke (Figure 2A). For the quantification of CD31^+^/BrdU^+^ cells, an investigator, who was blinded to the experimental groups, counted the number of CD31^+^/BrdU^+^ cells from 3 sections (200 μm before, center, and 200 μm after ischemic core) and 4 images from each brain section (upper, middle, and bottom of the peri‐ischemic region). Therefore, the cell counts from a total of 12 images were analyzed.

### 2.6 Western blot analysis

Protein was separately extracted from the regional brain tissues from −1.6 mm to +1.2 mm of the bregma of the ipsilateral hemisphere after MCAO and from different treated cells using protein RIPA lysis solution (Millipore), and protein content quantified with BCA protein assay (Thermo Fisher Scientific, Waltham, MA). The Western blot protocol was performed as previously described, and the primary antibodies were CD31 (1:5000 dilution, R&D systems), VEGF, VEGFR2, Tie2, Ang‐1, Ang‐2, bFGF, BDNF, GAPDH (1:5000 dilution, Proteintech Groups, Wuhan, China), and Shh (1:5000 dilution, Santa Cruz Biotechnology).^32^ An investigator who was blinded to the experimental groups measured the Western blot results.

### 2.7 Real‐time PCR

Regional brain tissues from −1.6 mm to +1.2 mm of the bregma of the ipsilateral hemisphere after MCAO were isolated using a TRIzol reagent (Invitrogen), and the expression of Shh was examined. Amplification was conducted by a real‐time PCR system (7900HT, ABI, CA) using a SYBR Premix Ex Taq Kit (Takara, Dalian, China).^33^ An investigator who was blinded to the experimental groups measured the results of the real‐time PCR. The primer sequences used in the experiment are as follows:

Shh: forward primer 5′‐TATGAGGGTCGAGCAGTGGA‐3′ and reverse primer 5′‐AGTGGATGCGAGCTTTGGAT‐3′.

GAPDH: forward primer 5′‐GATGGTGAAGGTCGGTGTGA‐3′ and reverse primer 5′‐TGAACTTGCCGTGGGTAGAG‐3′.

### 2.8 Synchrotron radiation angiography

The functional microvessel density of rats was assessed by a novel animal synchrotron radiation angiography (SRA) technique at the BL13W beamline in the Shanghai Synchrotron Radiation Facility (SSRF). The imaging setup and procedures for SRA imaging have been described previously.^34^ Briefly, 14 days after MCAO, rats were anesthetized by ketamine/xylazine (100 mg/10 mg/kg, intraperitoneally) and placed perpendicularly to the X‐ray beam during the imaging process. A PE‐10 tube was inserted into the common carotid artery, and iodinated contrast medium (Ipamiro, Shanghai, China) was injected through it as a bolus (160 μL at 100 μL/second) by using an automated microsyringe pump (LSP01‐1A, Longer, Baoding, China). The X‐ray energy was 33.3 keV, and an X‐ray complementary metal oxide semiconductor (pixel size of 6.5 × 6.5 μm, frame frequency of 50 Hz, Hamamatsu Ltd, Hamamatsu City, Japan) was used to record the high‐resolution real‐time angiographic images.

To assess the microvascular perfusion, MATLAB software (MathWorks, Natick, MA) was used with a short program, and an investigator who was blinded to the experimental groups identified the functional vessels from the original SRA images as described previously.^35^ Identical areas of the middle cerebral artery branches were selected, and a legible vessel map was obtained by denoising under the same threshold. The vessel density was defined as the ratio of pixels in vessels to the total pixels in each identical area.

### 2.9 Cell culture, siRNA transfection, and CM collection

Primary cortical astrocyte cultures were prepared from newborn Sprague Dawley rats (within 24 hours after birth) as described previously.^36^ After microglia and oligodendrocyte progenitors were removed, pure astrocytes were obtained and used for subsequent experiments.

Astrocytes were transfected with Shh‐specific small interfering RNA (si‐Shh) (GenePharma, Shanghai, China) using Lipofectamine^®^ 3000 transfection reagent (Invitrogen) and following the manufacturer's instructions. After the determination of the si‐Shh knockdown efficiency and concentration of NBP, the experiments in astrocytes were performed using four groups with different treatments: 0 μM NBP (blank); 10 μM NBP (NBP); si‐NC and 10 μM NBP (NC+NBP group); and si‐Shh and 10 μM NBP (si‐Shh+NBP).

NBP was dissolved in DMSO (Sigma, St. Louis, MO) before being diluted in the culture medium, and the final concentration of DMSO was 0.1%. The astrocytes were transfected with 50 nM siRNA for 48 hours and treated with NBP for 24 hours. Then, the CM of the four groups was removed and the cell samples were collected for Western blot analysis and immunostaining. The sequence of the Shh interference siRNA segment was 5′‐GUGGCACCAAGUUAGUGAATT‐3′.

### 2.10 Human umbilical cord vein cell proliferation assay

Human umbilical cord vein cells (HUVECs) were isolated as described previously and cultured using endothelial cell medium (Sciencell, San Diego, CA).^37^ HUVECs were seeded in 96‐well plates and incubated with the CM of the four astrocyte groups for 24 hours. Cell proliferation was determined by using immunostaining with the proliferation marker Ki‐67 (rabbit anti‐Ki‐67, Abcam, Cambridge, UK) as described previously.^28^ The Ki‐67‐positive cells were counted manually under a microscope, and each sample was observed in three random fields.

### 2.11 Wound healing assay

Human umbilical cord vein cells (5 × 10^5^ per well) were seeded into 12‐well plates and allowed to adhere for 24 hours. After using a 200‐μL pipette tip to establish the scratch wound model, the HUVECs were allowed to heal for 5 hours. Images were taken at the same site at 0 and 5 hours after the injury, and the healing of the wounds was assessed by measuring the reduction in the wound gap.^38^

### 2.12 Cell tube formation assay

A tube formation assay was carried out as previously described.^39^ Matrigel (BD Biosciences, Franklin Lakes, NJ) was added into a 96‐well plate and incubated at 37°C for 30 minutes to allow solidification. Then, 2 × 10^4^ HUVECs suspended in 30% CM of the four astrocyte groups and 70% endothelial cell medium were seeded into the coated plate in triplicate. After culturing for 3 hours, the tube images were captured by a microscope (Leica, Solms, Germany), and the tube numbers were counted by using ImageJ software.

### 2.13 Statistical analysis

Descriptive statistics are presented as the mean ± standard deviation. For comparisons between the data of two groups, a two‐tailed unpaired *t* test was used, and for multigroup comparisons, one‐tailed ANOVA followed by a post hoc Tukey test was used. For nonparametric analysis, the Mann‐Whitney *U* test was applied. Statistical analyses were performed using SPSS software (version 17.0, SPSS, Chicago, IL). A value of *P *< 0.05 was considered statistically significant.

## 3 RESULT

### 3.1 NBP attenuated brain infarct volume and neurological deficits following MCAO

**Figure 1:**
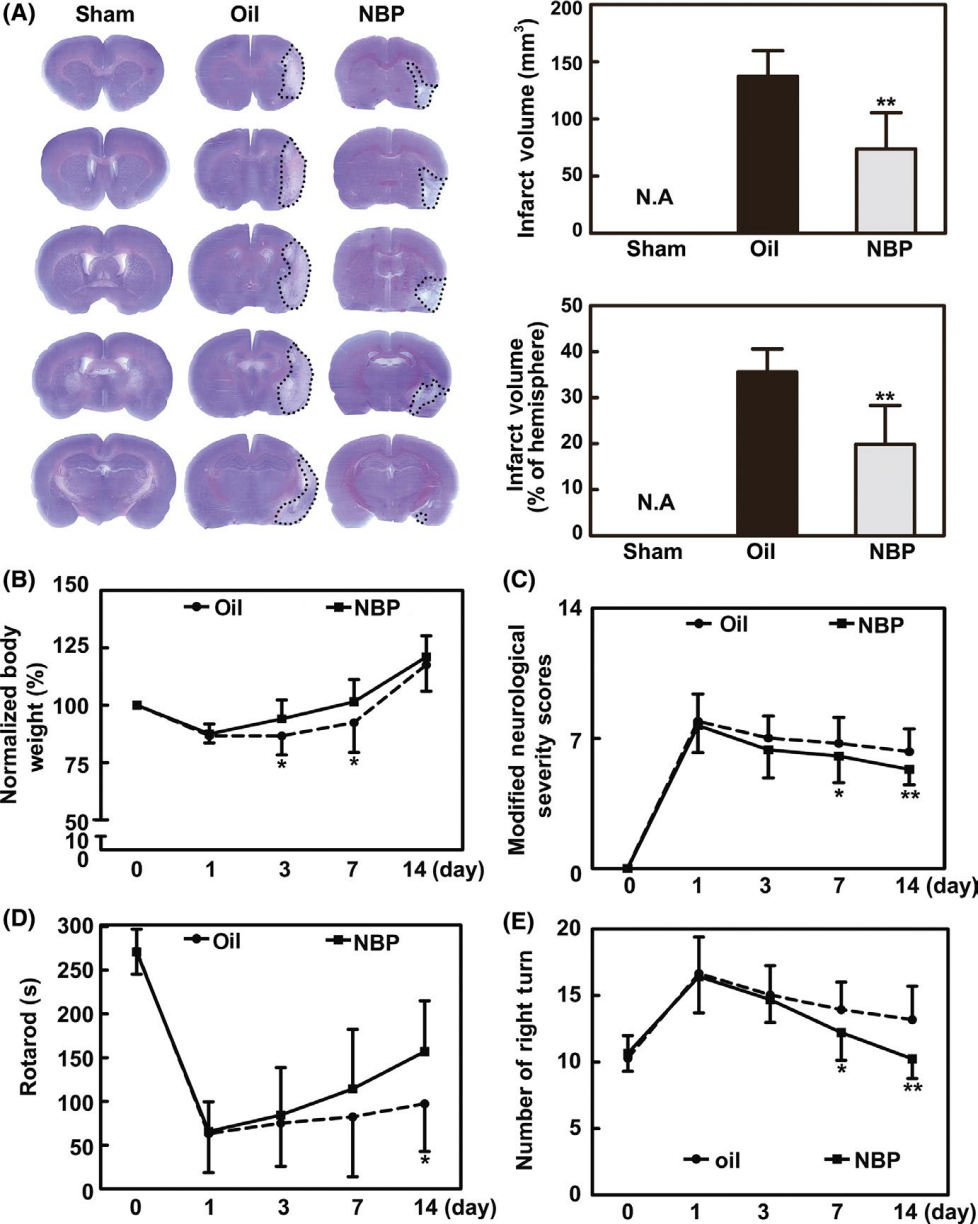
NBP treatment attenuated brain infarct volume and neurological deficits after MCAO. A, Brain infarction was detected by Cresyl violet staining 14 d after MCAO in the NBP‐treated and oil‐treated rats. The bar graphs show the statistical analysis of the infarct volume and proportion in the NBP‐treated and the oil‐treated rats. B, Body weight from day 0 to day 14, normalized to the original body weight, is presented. C‐E, Bar graphs summarizing the results of the mNSS evaluation, rotarod performance, and EBST test in the NBP‐treated and oil‐treated rats. n = 14‐16 per group. Data are presented as the mean ± SD, ^*^ *P < *0.05; ^**^ *P < *0.01, NBP‐treated vs oil‐treated rats

To determine whether NBP has a neuroprotective effect, we first examined infarct volume and neurobehavioral outcomes. We found that NBP significantly reduced brain infarct volume at 14 days and slowed the loss of body weight in the first 7 days after MCAO in rats (*P* < 0.05, Figure 1A,B). Similarly, NBP treatment significantly improved neurological outcomes 14 days after MCAO. The results of mNSS showed improved neurobehavioral outcomes from 7 to 14 days after MCAO in the NBP‐treated rats compared to the oil‐treated rats (*P* < 0.05, Figure 1C).

Rotarod tests, which are used for determining motor function, showed that the time on the rod was significantly longer in the NBP‐treated rats 14 days after MCAO (*P* < 0.05, Figure 1D). The EBST test showed that the asymmetric motor function was improved in the NBP‐treated rats from 7 to 14 days after MCAO (*P* < 0.05, Figure 1E). These results suggest that NBP has a neuroprotective effect during cerebral ischemia.

### 3.2 NBP promoted angiogenesis 14 days after MCAO

**Figure 2:**
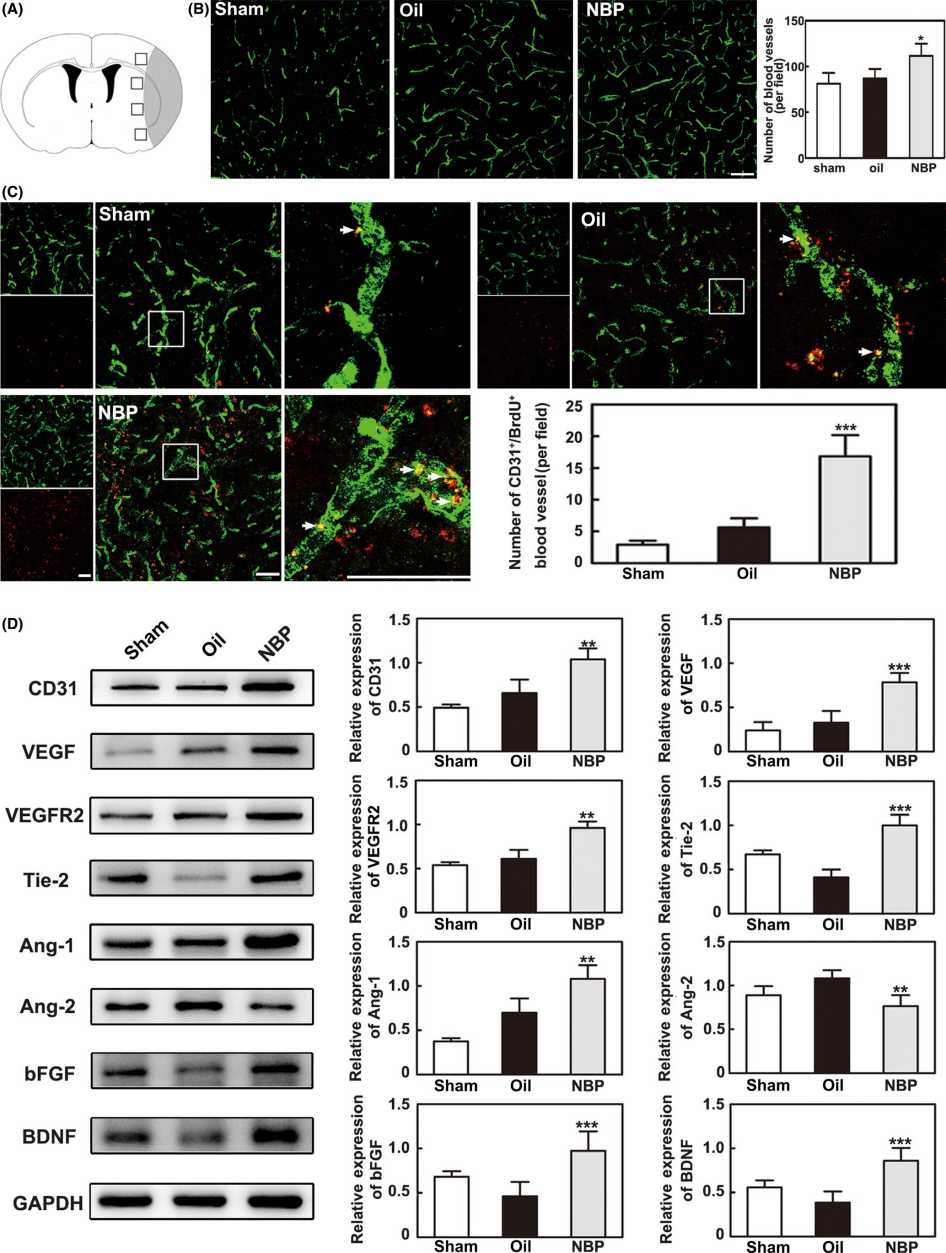
NBP promoted angiogenesis 14 d after MCAO. A, A schematic diagram of the immunostained images. The black boxes represent the areas of each section selected for imaging, and the gray area represents the infarcted brain regions. B, Representative images showing the immunostaining of CD31^+^ microvessels in the ipsilateral hemisphere after MCAO in the sham, oil, and NBP treatment groups. Bar = 100 μm, n = 6 per group. C, Representative images showing the immunostaining of CD31 (green) and BrdU (red) double‐stained cells in ischemic rats. Bar graphs show the quantification of the CD31^+^/BrdU^+^ cells in the NBP‐treated, oil‐treated, and sham groups. Bar = 50 μm, n = 6 per group. D, Western blot showing angiogenic growth factor expression in the ischemic rat brain 14 d after MCAO. Bar graphs show the data quantification. n = 8 per group. Data are presented as the mean ± SD, ^*^ *P < *0.05; ^**^ *P < *0.01; ^***^ *P < *0.001, NBP‐treated vs oil‐treated rats

CD31^+^ endothelial cells are used to evaluate the number of microvessels with immunostaining and are one method to detect angiogenesis.^40^ To determine whether NBP promoted angiogenesis in the peri‐infarct area, the number of CD31^+^ microvessels was examined in the ischemic perifocal region 14 days after MCAO. Immunostaining results showed that the number of CD31^+^ cells increased in the ischemic perifocal region of NBP‐treated rats compared to that of the oil‐treated rats (*P* < 0.05, Figure 2B). It was noted that NBP treatment increased the number of CD31^+^/BrdU^+^ cells in the peri‐infarct region compared to the oil‐treated rats (*P* < 0.001, Figure 2C), suggesting an effect of NBP on endothelial cell proliferation.

To further detect the factors that induced the increase in CD31^+^ microvessels, angiogenic growth factor expression in the ischemic brain was examined by Western blot analysis 14 days after MCAO. We found that the protein levels of CD31, VEGF, VEGFR2, Ang‐1, Tie2, bFGF, and BDNF were significantly increased in the NBP‐treated rats compared to the oil‐treated rats (*P* < 0.01, Figure 2D). Interestingly, Ang‐2 expression decreased, which was the opposite of the effect on Ang‐1 at 14 days.

**Figure 3:**
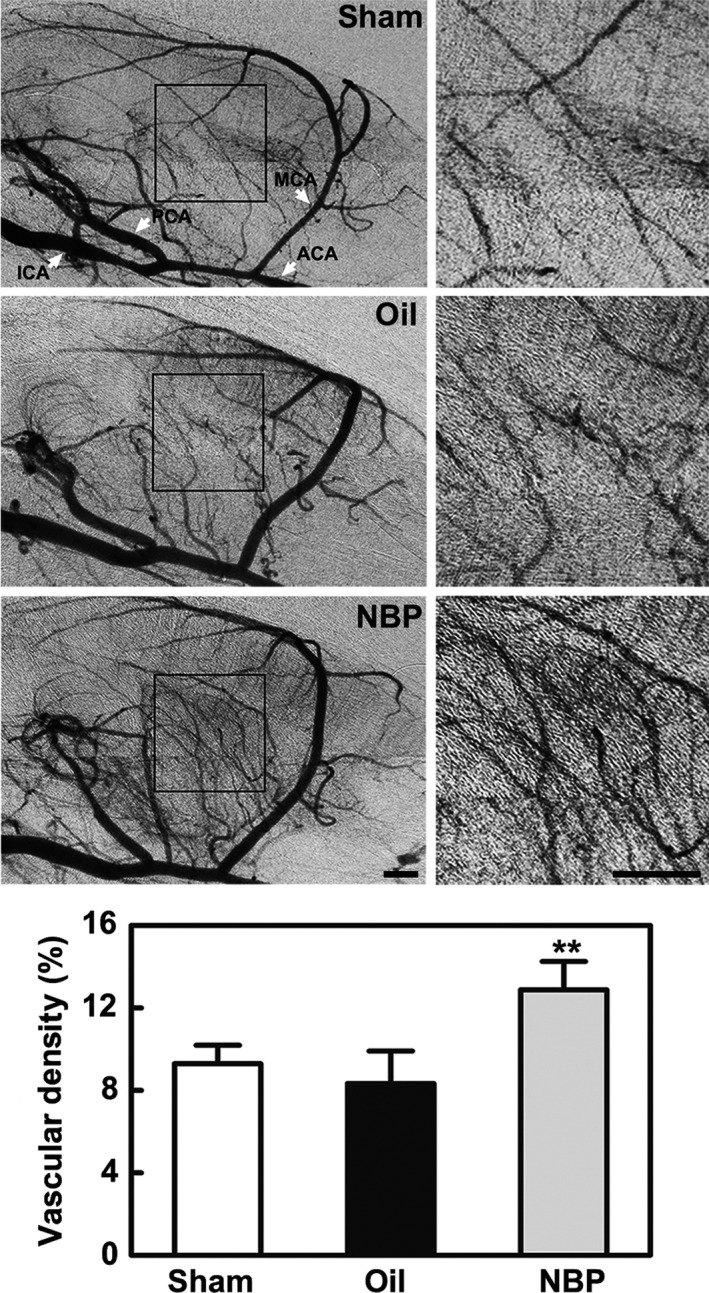
NBP treatment increased the perfused small vessel density in ischemic rats. Black boxes show the area used for measurement. Bar graphs show the vascular density in the NBP‐treated, oil‐treated, and sham groups 14 d after middle cerebral artery occlusion. ACA, anterior cerebral artery; ICA, internal carotid artery; MCA, middle cerebral artery; PCA, posterior cerebral artery. Bar = 1 mm, n = 8 per group. Data are presented as the mean ± SD, ^**^ *P < *0.01, NBP‐treated vs oil‐treated rats

To determine whether the increased microvessels were functional, the perfused vascular density in the ischemic peri‐infarct region was calculated from the original SRA images. The perfused vessel density of NBP‐treated rats was much higher than that of the oil‐treated rats (*P* < 0.01, Figure 3). Taken together, the results indicated that NBP treatment promoted angiogenesis of the peri‐infarct region after 14 days of MCAO.

### 3.3 NBP upregulated Shh expression in astrocytes in vivo and in vitro

**Figure 4:**
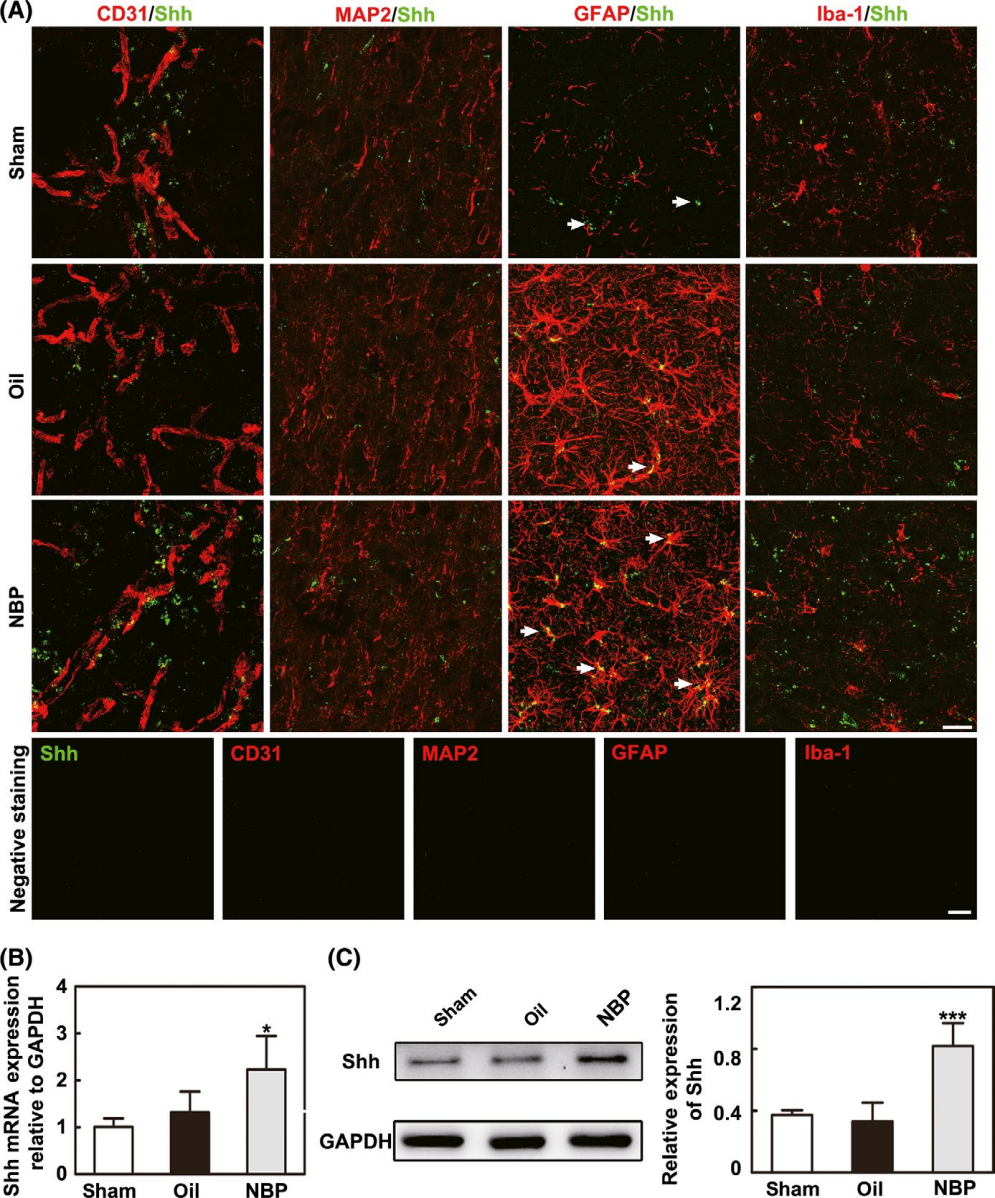
NBP upregulated the expression of Shh in astrocytes in vivo. A, Representative images showing the immunostaining of CD31, MAP2, GFAP, and Iba‐1 (red) with Shh (green) and the negative control staining in the ipsilateral hemisphere after middle cerebral artery occlusion (MCAO) in the sham, oil, and NBP treatment groups. Bar = 25 μm, n = 6 per group. B, The results of the real‐time PCR show Shh expression in the NBP‐treated, oil‐treated, and sham groups after 14 d of MCAO. n = 8 per group. C, Western blot shows the expression of Shh in the sham, oil, and NBP‐treated groups 14 d after MCAO. Bar graphs show the quantification of Shh expression. n = 8 per group. Data are presented as the mean ± SD, ^*^ *P < *0.05; ^***^ *P < *0.001, NBP‐treated vs oil‐treated rats

Double immunostaining of CD31^+^/Shh^+^, MAP2^+^/Shh^+^, GFAP^+^/Shh^+^, and Iba1^+^/Shh^+^ showed that Shh was mainly expressed in astrocytes, not in endothelial cells, neurons, or microglia. We found that the level of Shh was upregulated in the NBP‐treated rats 14 days after MCAO, and the negative control for the staining demonstrated that the immune signal was specific (*P* < 0.05, Figure 4A). Real‐time PCR and Western blot analysis also showed that Shh expression was upregulated in the NBP‐treated rats compared to the oil‐treated rats 14 days after MCAO (*P* < 0.05, Figure 4B,C).

**Figure 5:**
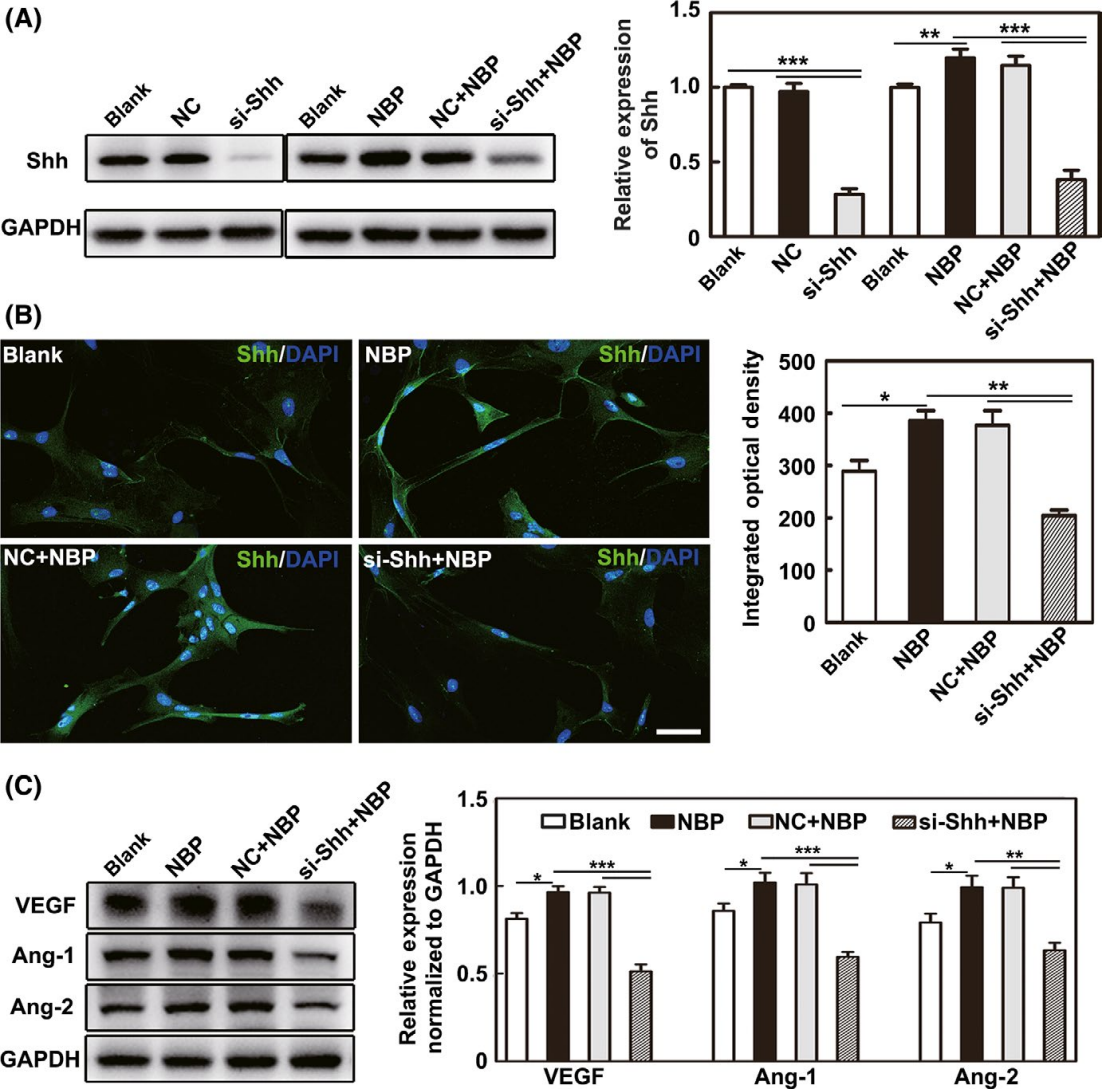
NBP upregulated Shh expression in astrocytes and further increased VEGF, Ang‐1 and Ang‐2 expression in HUVECs. A, Western blot images and data quantification show the Shh expression in the different astrocyte treatment groups. B, Representative images show the immunostaining for Shh (green) and DAPI (blue) in astrocytes. Bar graphs show the quantification of Shh. Bar = 50 μm. C, Western blot shows VEGF, Ang‐1, and Ang‐2 expression in HUVECs after treatment with the CM of astrocytes. Bar graphs show the quantification data. n = 3 per group. Data are presented as the mean ± SD, ^*^ *P < *0.05; ^**^ *P < *0.01; ^***^ *P < *0.001

We also detected Shh expression in cultured astrocytes, and Western blot results showed that Shh was significantly higher in the NBP‐treated astrocytes than in the oil‐treated astrocytes (*P* < 0.01), while siRNA Shh interference significantly decreased Shh expression in astrocytes (*P* < 0.001, Figure 5A,B).

### 3.4 Shh promoted VEGF, Ang‐1, Ang‐2 expression, and HUVEC proliferation, migration, tube formation

After HUVECs were incubated with the CM of the different astrocyte treatment groups, the expression of VEGF, Ang‐1, and Ang‐2 was examined. Western blot analysis showed that the expression of VEGF, Ang‐1, and Ang‐2 was significantly higher in the NBP‐treated group than in the blank group and the siRNA Shh interference significantly decreased Shh expression in the si‐Shh+NBP group compared to the NC+NBP group (*P* < 0.05, Figure 5C).

**Figure 6:**
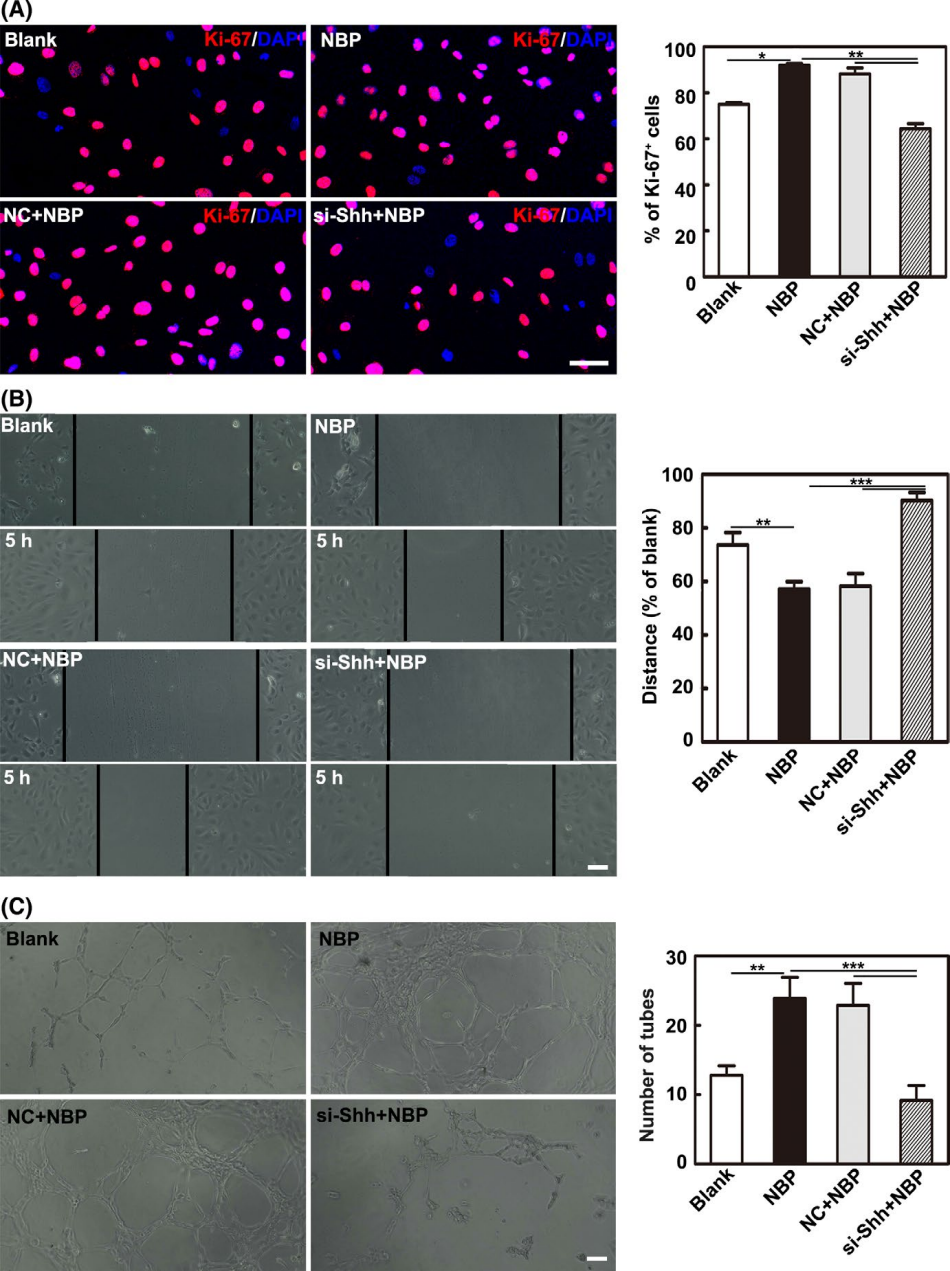
Shh improved proliferation, migration, and tube formation of HUVECs. A, Images and bar graph show the cell proliferation ability of HUVECs after treatment with the CM of astrocytes. Bar = 50 μm. B, Representative images and bar graph show cell migration ability of HUVECs after treatment with the CM of astrocytes. Bar = 100 μm. C, Representative images and bar graph show the tube formation ability of HUVECs after treatment with the culture medium of astrocytes. Bar = 100 μm. n = 3 per group. Data are presented as the mean ± SD, ^*^ *P < *0.05; ^**^ *P < *0.01; ^***^ *P < *0.001

To examine the direct effect of NBP on endothelial cells via Shh, HUVEC proliferation, migration, and tube formation were analyzed. We demonstrated that the NBP treatment promoted HUVEC proliferation, migration, and tube formation while Shh knockdown with siRNA reversed these effects (*P* < 0.05, Figure 6A‐C).

## 4 DISCUSSION

In the present study, we explored the effect of the novel drug NBP on the promotion of angiogenesis during cerebral ischemia. We examined the effect of NBP on the promotion of angiogenesis using in vitro and in vivo models. We demonstrated that (a) NBP treatment could attenuate brain infarct volume and neurological deficits in MCAO rats; (b) NBP treatment could increase the expression of the angiogenic growth factors VEGF and Ang‐1, consequently promoting angiogenesis in the peri‐infarct area; and (c) the pro‐angiogenic effect of NBP was associated with the increased expression of Shh in astrocytes. Our results suggested that NBP was not only beneficial for acute stroke therapy but was also potentially favorable for neurological repair and remodeling following MCAO.

Angiogenesis is an important step in postischemic recovery. Several factors, such as VEGF, Ang‐1, and bFGF, could promote angiogenesis in the brain during cerebral ischemia.^41^, ^42^ Previous studies demonstrated that NBP could promote angiogenesis.^38^, ^39^ The incidence of ischemic stroke and the infarct volume were decreased with NBP pretreatment in stroke‐prone renovascular hypertensive rats.^22^, ^43^ In addition, NBP treatment within 24 hours after ischemic stroke rescued brain tissue by enhancing angiogenesis or opening collateral vessels, which was associated with the upregulation of VEGF and HIF‐1α expression, in the stroke‐prone renovascular hypertensive rats.^22^ An experiment demonstrated that angiogenesis was more vigorous after NBP treatment and that the expression of the growth factors including VEGF, VEGF receptor, and bFGF was increased in the chick embryonic chorioallantoic membrane assay.^23^ We found that NBP not only exhibited neuroprotection in the acute phase of ischemic stroke but also promoted angiogenesis in the later phase of ischemic stroke, which is an extremely important step for long‐term neurobehavioral recovery. A strength of NBP in the promotion of angiogenesis appears to be targeting multiple elements, which is better than other treatments.^13^ Our results supported the notion that NBP promoted angiogenesis after ischemic stroke because of the following: (a) NBP increased the number of CD31^+^ microvessels and the number of CD31^+^/BrdU^+^ cells; (b) NBP promoted proliferation, migration, and tube formation in HUVECs in vitro; (c) NBP could increase the expression of several families of growth factors, such as VEGF, Ang‐1, and bFGF, both in vivo and in vitro; and (d) NBP increased the functional vascular density in the peri‐infarct regions of ischemic rat brain.

The molecular mechanism of NBP in the promotion of angiogenesis is largely unknown. We found that the effect of NBP appeared to be related to Shh. The role of Shh in the development of functional vasculature has been observed for various tissues.^44^, ^45^, ^46^ Shh acts on upstream of the angiogenic growth factors VEGF and angiopoietins and can particularly promote the upregulation of VEGF and angiopoietin‐1 expression.^47^, ^48^ Angiogenic growth factors induce and promote the steps of angiogenesis and play a significant role in cell proliferation, maturation, and differentiation, leading to the formation of mature blood vessels.^49^, ^50^ The administration of a Shh‐neutralizing antibody inhibited angiogenesis and VEGF upregulation in mouse ischemic legs, implying a role for endogenous Shh signaling in ischemic angiogenesis.^51^ In addition, Shh promotes the neovascularization of ischemic tissues by promoting angiogenesis when administered either as a recombinant protein or via gene therapy.^48^, ^52^ Shh is one of the key elements that guides angiogenesis.^51^ Our study demonstrated that astrocytic Shh expression was upregulated in the NBP‐treated rats and that downstream proteins such as VEGF, Ang‐1, and bFGF were also increased. These angiogenic growth factors are essential to promoting angiogenesis and neurological repair and remodeling. This effect could be reversed when Shh is inhibited in vitro. It was noted that astrocytes could secrete Shh.^21^ In our immunostaining study, we found that Shh was mainly expressed in astrocytes, not in endothelial cells, neurons, or microglia, suggesting that astrocytes were a main source of Shh, which further regulated VEGF and Ang‐1 expression and eventually promoted angiogenesis.

We used a novel SRA technique to quickly and dynamically semiquantify angiogenesis in living animals. Several methods have been used to detect angiogenesis. However, most methods only study brain sections and not living animals. Laser Doppler or two‐photon microscopy could detect angiogenesis in living animals. However, only the surface blood flow can be detected, and the image window is very limited.^34^ The immunohistochemical staining of cerebral blood vessels could only reflect the number of blood vessels, not the exact number of the perfused microvessels.^35^ The spatial resolution of SRA can reach submicrons, approximately 1000 times higher than that of conventional X‐ray absorption imaging, and it can be used for high‐resolution imaging of the whole middle cerebral artery territory blood supply in living rodents.^53^, ^54^ Vessel density is an indicator of microvascular perfusion. Our results showed that NBP treatment increased the perfused vessel density. SRA is a better way to study cerebral microcirculation. The novel SRA technique provides a unique tool for monitoring real‐time hemodynamic changes in blood flow and microvascular morphology.

In summary, our study was the first to systematically demonstrate that NBP treatment improved angiogenesis by upregulating astrocyte Shh expression in the ischemic brain. Shh then further promoted the expression of the angiogenic factors VEGF and Ang‐1. This result suggested that NBP could not only be used as a neuroprotective drug in the acute phase of ischemic stroke but could also be used to promote neurological repair and remodeling drug in the later phase of ischemic stroke.

## CONFLICT OF INTEREST

The authors declare no conflict of interest.
